# First-principles exploration of the electronic versatility of the GeH/SiSb heterostructure through stacking and electric fields†

**DOI:** 10.1039/d5na00251f

**Published:** 2025-05-21

**Authors:** Nguyen Xuan Sang, Khang D. Pham

**Affiliations:** a Atomic Molecular and Optical Physics Research Group, Institute for Advanced Study in Technology, Ton Duc Thang University Ho Chi Minh City Vietnam nguyenxuansang@tdtu.edu.vn; b Faculty of Electrical and Electronics Engineering, Ton Duc Thang University Ho Chi Minh City Vietnam; c Institute of Research and Development, Duy Tan University Da Nang 550000 Vietnam phamdinhkhang@duytan.edu.vn; d School of Engineering & Technology, Duy Tan University Da Nang 550000 Vietnam

## Abstract

Designing heterostructures is crucial for developing advanced materials with tailored properties for specific applications. In this study, we explored the intrinsic properties, stability, and tunability of the GeH/SiSb heterostructure through various stacking patterns and the application of electric fields. Our findings confirm that combining GeH and SiSb monolayers creates a stable heterostructure, as evidenced by phononic spectrum analysis, AIMD simulations, and mechanical property evaluations. Depending on the stacking patterns, the heterostructure exhibits either type-I or type-II band alignments. Additionally, the application of electric fields effectively modulates the band gap, facilitates transitions between type-I and type-II alignments, and transforms the band gap from indirect to direct. These findings underscore the versatility of the GeH/SiSb heterostructure for next-generation optoelectronic devices, offering precise electronic property control to enhance device performance.

## Abstract 2

Designing heterostructures is crucial for developing advanced materials with tailored properties for specific applications.

## Graphical abstract

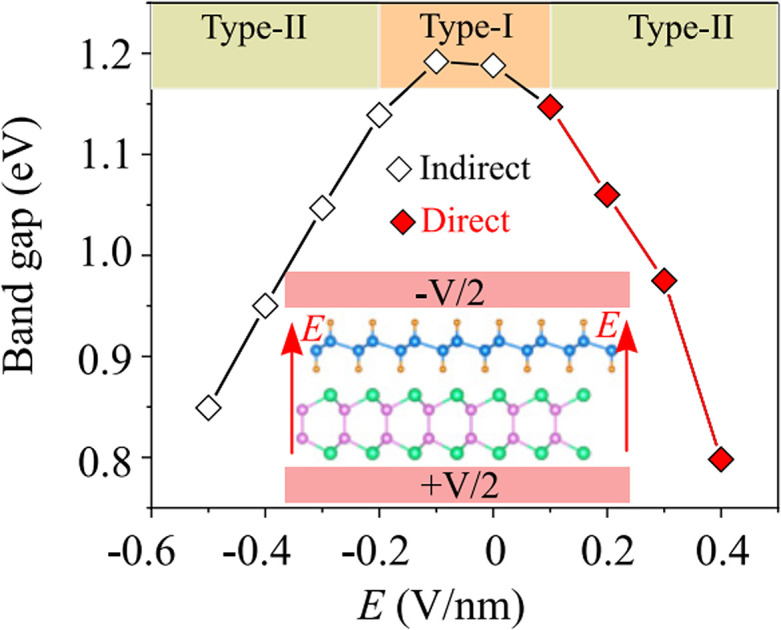

## 1 Introduction

The discovery of graphene^1^ in 2004 marked a significant breakthrough in materials science, paving the way for a new era for two-dimensional (2D) materials. Graphene, a single layer of carbon atoms arranged in a honeycomb lattice, demonstrated extraordinary properties, sparking extensive research in the realm of 2D materials.^2^ Following graphene, a diverse array of 2D materials has been synthesized, including transition metal dichalcogenides (TMDs),^3^ black phosphorus,^4^ and MXenes.^5^ These materials have demonstrated potential in a range of applications, from flexible and transparent electronics^6,7^ to advanced energy storage systems such as high-performance batteries^8^ and next-generation semiconductor devices.^9^

Recently, germanane (GeH) has gained attention as a promising 2D material, discovered through the experimental topochemical deintercalation of CaGe_2_ in aqueous HCl, resulting in a stable, hydrogenated monolayer of germanium.^10^ The material exhibits intriguing electronic characteristics, such as high charge carrier mobility^11,12^ and controllable electronic and optical properties^13,14^ that make it an attractive candidate for applications in optoelectronics and electronics. Similarly, the silicon antimonide (SiSb) monolayer is another emerging 2D material that has attracted significant attention due to its unique properties and potential applications. The SiSb monolayer exhibits a honeycomb-like crystal structure similar to other 2D materials.^15^ First-principles calculations have revealed that the SiSb monolayer possesses a direct band gap, making it suitable for optoelectronic applications such as photodetectors, light-emitting diodes (LEDs), and solar cells.^16,17^ It also exhibits excellent thermal stability, essential for the reliability and longevity of devices operating at elevated temperatures.^16^ The electronic properties of the SiSb monolayer can be finely tuned through various external stimuli,^18^ making it highly versatile for practical applications. For instance, applying mechanical strain can significantly alter the band gap and charge carrier distribution of the SiSb monolayer,^15^ enabling precise control over its electronic behavior. This tunability is valuable for designing electronic and optoelectronic devices.

Despite the remarkable properties of individual 2D materials, there is a growing interest in constructing heterostructures^19,20^ – the combinations of different 2D layers stacked together. These heterostructures enable the engineering of new materials with tailored properties that are not achievable with single-component systems.^21–25^ As a result, 2D heterostructures have become a key focus in contemporary materials research, driving advancements in nanoelectronics, optoelectronics, and energy storage.^26,27^ To date, various heterostructures based on GeH monolayers have been discovered.^28–32^ The integration of GeH with other 2D materials induces novel phenomena that are absent in the isolated GeH monolayer. For instance, Jin *et al.*^29^ demonstrated that combining GeH with graphene facilitates the separation of photogenerated carriers and enhances optical absorption, making it a promising candidate for optoelectronic applications. Furthermore, using first-principles predictions, Li *et al.*^30^ revealed that the GeH/InSe heterostructure forms a Z-scheme configuration, which is highly beneficial for overall photocatalytic water splitting. In this work, using first-principles prediction, we investigated the GeH/SiSb heterostructure, focusing on its intrinsic properties, stability, and tunability under different stacking patterns and electric fields. Our results reveal that the GeH/SiSb heterostructure can form either type-I or type-II band alignments with highly tunable electronic properties and contact behavior. The application of an electric field induces transitions between type-I and type-II band alignments, and shift the band gap from indirect to direct. Our insights highlight the significant potential of the GeH/SiSb heterostructure in next-generation optoelectronic applications.

## 2 Computational methods

We performed first-principles calculations using the Quantum Espresso package^33^ based on density functional theory (DFT). The exchange-correlation potential was treated with the Perdew–Burke–Ernzerhof (PBE)^34^ functional in the framework of the generalized gradient approximation (GGA). To ensure accurate electronic structure calculations, we employed the Heyd–Scuseria–Ernzerhof (HSE06) hybrid functional^35^ for band gap corrections. The projector-augmented wave (PAW) method^36^ was utilized to describe the electron–ion interactions. A kinetic energy cutoff of 500 eV was set for the plane-wave basis set and the Brillouin zone integration was carried out using a Γ-centered Monkhorst–Pack *k*-point mesh of 9 × 9 × 1.^30,31,37^ The lattice parameters and atomic positions were fully relaxed until the forces on each atom were less than 0.01 eV Å^−1^ and the energy convergence criterion was set to 10^−6^ eV. The DFT-D3 Grimme method^38^ was employed to account for the weak vdW interactions in the GeH/SiSb heterostructure. *Ab initio* molecular dynamics (AIMD) simulations were also performed to evaluate the thermal stability of the heterostructure. These simulations were conducted at room temperature for a duration of 8 ps with a time step of 1 fs. The phonon dispersion was calculated using density functional perturbation theory (DFPT)^39^ as implemented in the Phonopy package.

## 3 Results and discussion

**Fig. 1:**
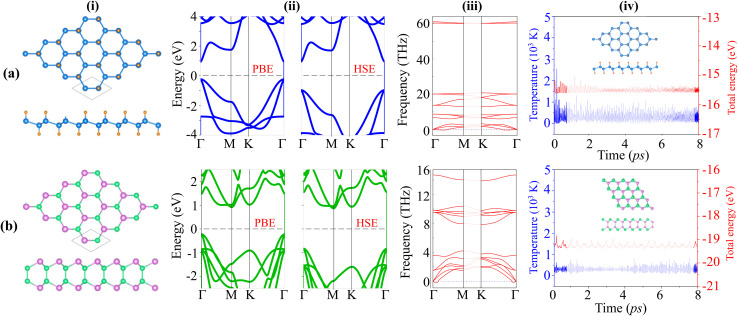
(i) Atomic structures, (ii) band structures, (iii) phonon spectra and (iv) AIMD simulations of the (a) GeH and (b) SiSb monolayers. Blue and orange balls stand for the Ge and H atoms, respectively, while the purple and green balls represent the Si and Sb atoms, respectively.

To start, we examine the intrinsic characteristics of GeH and SiSb monolayers, including their lattice parameters, electronic properties, and stability. Both GeH and SiSb monolayers exhibit hexagonal lattice structures. The GeH monolayer has a buckled structure, with each unit cell consisting of four atoms: two germanium (Ge) atoms that are buckled and sandwiched between two hydrogen (H) atoms. In contrast, the SiSb monolayer features a sandwiched structure, with its unit cell also containing four atoms: two antimony (Sb) atoms layered between two silicon (Si) atoms, as illustrated in Fig. 1(i). For the GeH monolayer, the lattice parameters are derived from its hexagonal crystal structure, typically exhibiting a lattice constant around 4.03 Å. This value aligns well with previous reports.^28,30^ Similarly, the SiSb monolayer exhibits a hexagonal crystal structure with a slightly smaller lattice constant of 3.97 Å, consistent with existing literature.^17^ Both the GeH and SiSb monolayers exhibit semiconducting behavior, as displayed in Fig. 1(ii). The GeH monolayer features a direct band gap, which is beneficial for optoelectronic applications due to efficient electron–hole recombination. The band edges of the GeH monolayer are located at the *Γ* point. In contrast, the SiSb monolayer demonstrates an indirect band gap, where the conduction band minimum (CBM) and valence band maximum (VBM) are at different points of *Γ* and *M* points in the Brillouin zone (BZ). The calculated band gap of the GeH (SiSb) monolayer is 1.15/1.89 (1.13/1.69) eV for the PBE/HSE calculation. These values are comparable with those obtained in previous reports.^16,28,30^ Both PBE and HSE calculations exhibit consistent behavior for the GeH and SiSb monolayers, validating our approach. Given the alignment of results and to optimize computational resources, all subsequent calculations will be conducted using the PBE method. Furthermore, we examine the stability of these monolayers by establishing their phonon spectra and AIMD simulations, as illustrated in Fig. 1(iii) and (iv). The phonon dispersion curves of the GeH and SiSb monolayers exhibit positive frequencies at the *Γ* point in the Brillouin zone, indicating their dynamic stability. Furthermore, AIMD simulations show small fluctuations in the total energy and temperature of these monolayers, with their atomic structures remaining intact after heating up to 8 ps. These findings confirm that GeH and SiSb monolayers are thermally stable at room temperature.

**Fig. 2:**
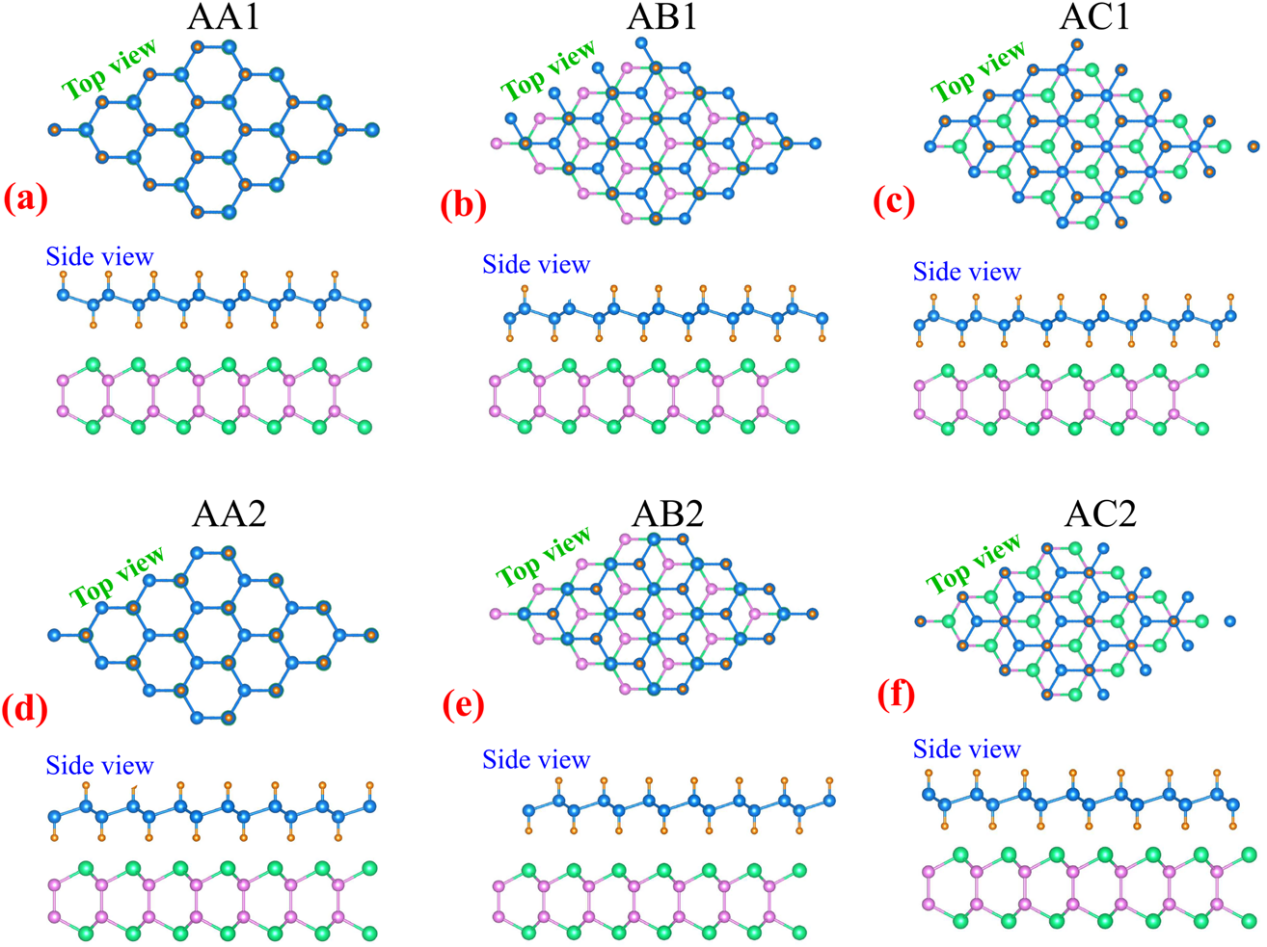
The atomic structures of six different stacking configurations of the GeH/SiSb heterostructure, including (a) AA1, (b) AB1, (c) AC1, (d) AA2, (e) AB2 and (f) AC2.

**Fig. 3:**
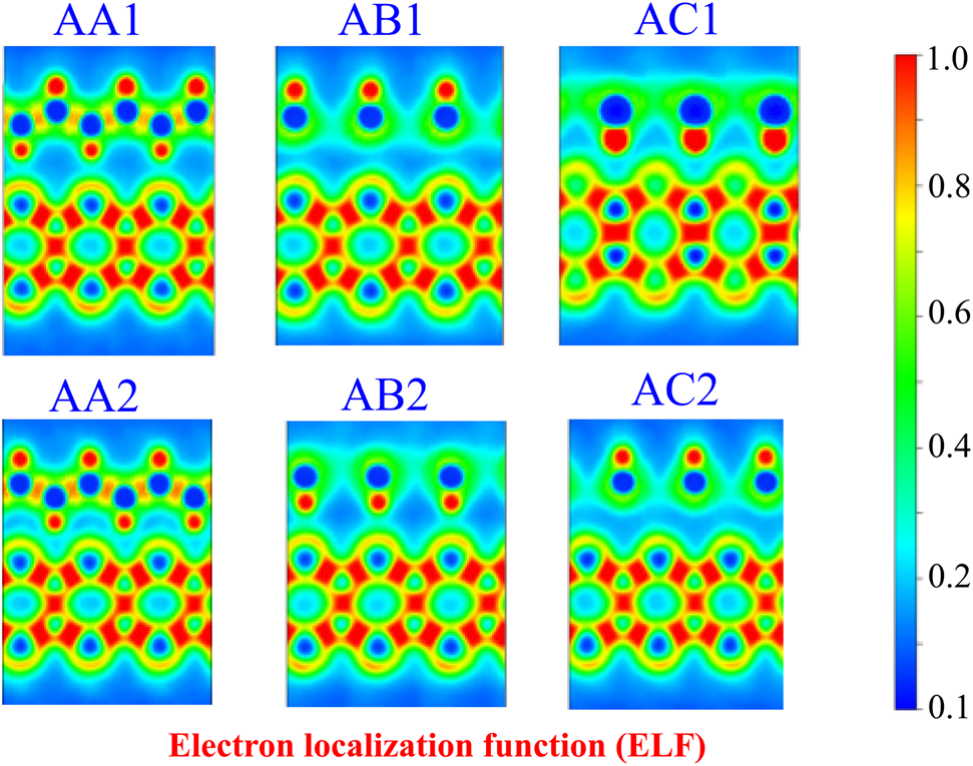
The visualization of the electron localization function (ELF) in the GeH/SiSb heterostructure for all patterns. Electrons are fully localized when the ELF equals 1.

Combining two different 2D materials often leads to lattice mismatches, which can give rise to various possible configurations of heterostructures. In the case of GeH and SiSb monolayers, this combination gives rise to six distinct configurations, namely AA1, AB1, AC1, AA2, AB2 and AC2, as depicted in Fig. 2. The lattice constant of the GeH/SiSb heterostructure is calculated to be 4.0 Å across all stacking patterns. This results in a lattice mismatch of just 0.8%, which is sufficiently small to be considered negligible. This minimal mismatch indicates that the GeH/SiSb heterostructure retains excellent structural compatibility and stability. In the AA stacking patterns, the Ge–H layers of the GeH monolayer are directly aligned above the Si and Sb layers of the SiSb monolayer. For the AB stacking patterns, one Ge–H layer is positioned above the Sb layer, while the other layer is centered over a hexagonal Si–Sb ring. In the AC stacking patterns, one Ge–H layer is situated above the Si layer, with the other layer centered over a hexagonal Si–Sb ring. The obtained interlayer distance *d* between the topmost Sb layer of the SiSb layer and the lowest H layer in the GeH layer for all patterns is listed in Table 1. The lowest *d* is observed for the AB2 pattern, while the AA1 pattern exhibits the highest *d*. The obtained *d* varies from 2 to 3 Å, which is the same as that obtained in other GeH-based heterostructures.^28–30^ Additionally, one can find that the obtained *d* is much higher than the bond length between H and Sb atoms (about 1.74 Å), suggesting that the interactions between the GeH and SiSb layers are mainly dominated by weak van der Waals (vdW) forces. Furthermore, to detail the interactions between the GeH and SiSb layers, we visualize the electron localization function (ELF) of the GeH/SiSb heterostructure across all configurations, as shown in Fig. 3. Electrons are fully localized when the ELF equals 1, whereas values approaching zero indicate regions with low electron density. The ELF at the interface of the GeH/SiSb heterostructure is evidently delocalized, suggesting the absence of chemical bonding between the two layers. Further ELF analysis confirms the lack of covalent bonds between the H and Sb layers, confirming that the GeH/SiSb heterostructure is governed by weak vdW interactions rather than covalent bonding.

**Table 1:** Calculated interlayer distance (*d*, Å), binding energy (*E*_b_, meV Å^−2^), band gap (*E*_g_, eV) and contact types of the GeH/SiSb heterostructure for six different patterns

Patterns	*d*	*E*_b_	*E*_g_	Types
AA1	3.01	−7.80	1.00	Type-II
AB1	2.11	−12.93	1.19	Type-I
AC1	2.18	−12.42	1.12	Type-II
AA2	3.00	−7.82	1.10	Type-I
AB2	2.05	−12.48	0.99	Type-II
AC2	2.19	−12.81	1.20	Type-I

**Fig. 4:**
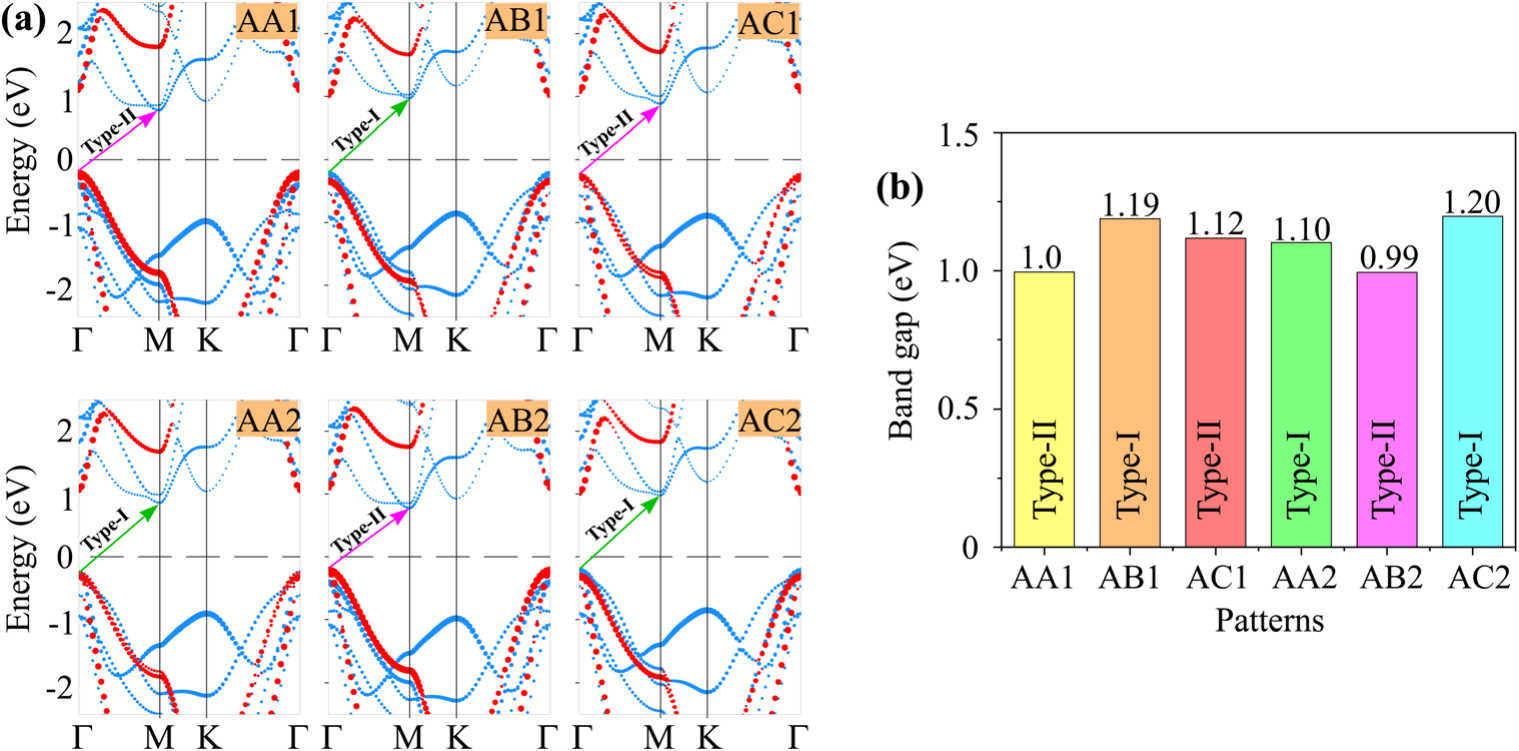
(a) The weighted projections of the band structures and (b) band gaps of the GeH/SiSb heterostructure for all patterns. The weighted projections of the GeH and SiSb layers are denoted by red and blue circles, respectively. Green and purple arrows represent the type-I and type-II band alignments of the heterostructure.

**Fig. 5:**
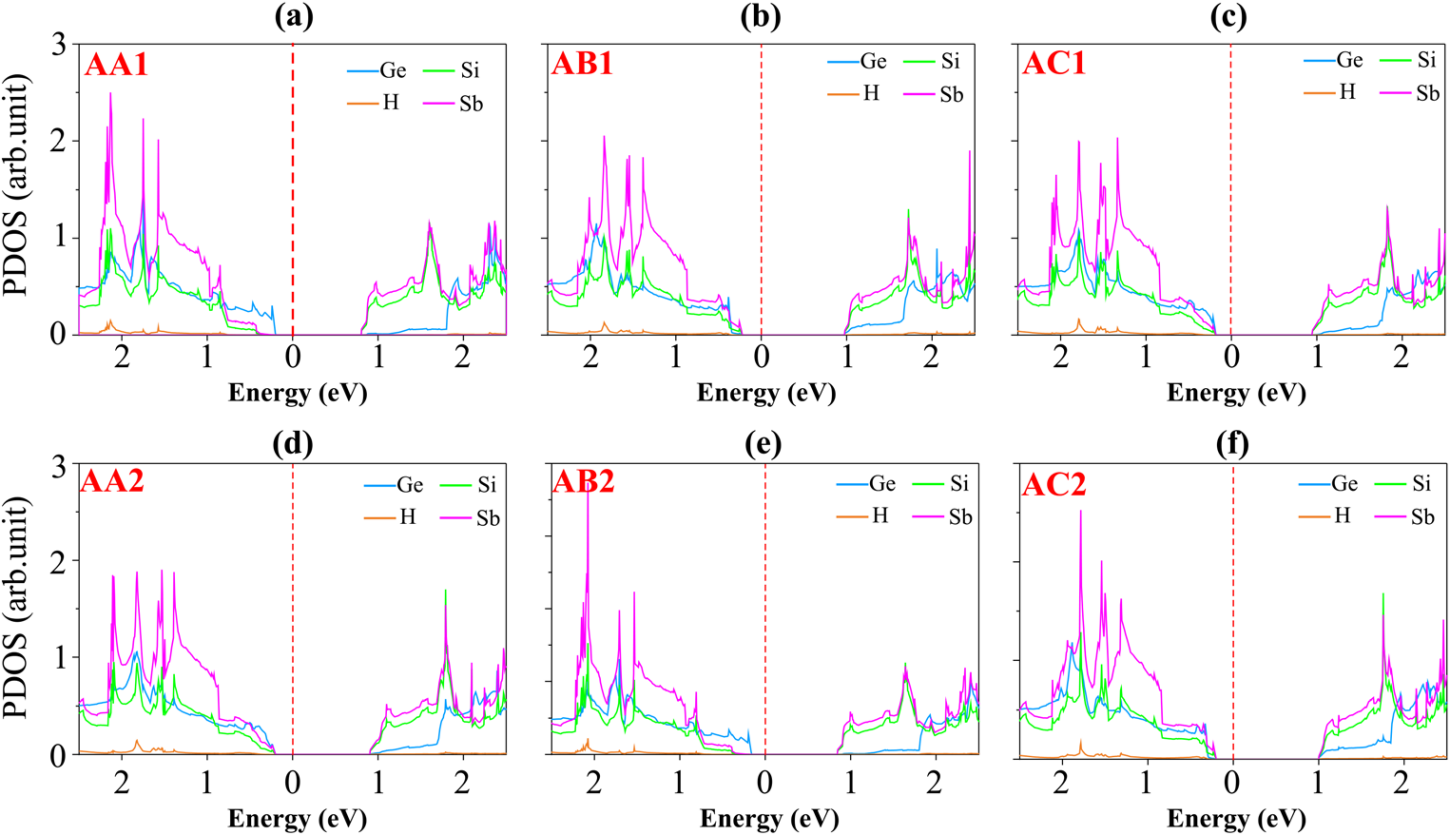
PDOS of all atoms in the GeH/SiSb heterostructure for (a) AA1, (b) AB1, (c) AC1, (d) AA2, (e) AB2 and (f) AC2.

Fig. 4 illustrates the weighted projections of the band structures of the GeH/SiSb heterostructure for all stacking patterns. All patterns display semiconducting behavior with indirect band gaps. The calculated PBE band gaps, listed in Table 1 and shown in Fig. 4(b), range from 0.99 to 1.20 eV, varying with the stacking patterns. This variation indicates that different stacking configurations can alter the band gaps of the heterostructure. According to the Anderson rule, the integration of 2D semiconductors can induce the formation of type-I, type-II or type-III band alignments.^40,41^ Additionally, we observe that the GeH/SiSb heterostructure can form either type-I or type-II band alignments, depending on the specific stacking pattern. For the AA1 patterns, the VBM is dominated by the GeH layer, while the CBM at the M point involves the SiSb layer. This is further corroborated by the partial density of states (PDOS) analysis of all atoms in the AA1 pattern, as shown in Fig. 5(a). The PDOS reveals that the VBM of the GeH/SiSb heterostructure originates from the Ge atoms of the GeH layer, whereas the CBM is due to the hybridizations between the Si and Sb atoms in the SiSb layer. This observation indicates the formation of a type-II band alignment in the AA1 pattern. The type-II band alignment is also present in the AC1 and AB2 patterns. The PDOS for all atoms in the AC1 and AB2 patterns, displayed in Fig. 5(c) and (e), further confirms the formation of the type-II band alignment in these configurations. For the AB1 pattern, the VBM and CBM at the *Γ* and *M* points are due to the contributions of the SiSb layer, specifying the formation of a type-I band alignment. The PDOS for all atoms in the AB1 pattern is illustrated in Fig. 5(b). One can find that the VBM and CBM in the AB1 pattern originate from the hybridizations between the Si and Sb atoms in the SiSb layer. This evidence confirms the formation of the type-I band alignment in the AB1 pattern of the GeH/SiSb heterostructure. Furthermore, the projected band structure of the AB1 pattern obtained using the HSE method is presented in Fig. S1 of the ESI.† The HSE band gap of the AB1 pattern is determined to be 1.78 eV, which is larger than the value predicted by the PBE method (1.19 eV). However, both PBE and HSE calculations predict the same type-I contact behavior for the AB1 pattern. A similar observation was also predicted in the GeH/InSe heterostructure.^30^ Hence, the PBE method serves as a reliable approach for predicting the contact behavior of the GeH/SiSb heterostructure, which is the main focus of our study. Similarly, the AA2 and AC2 patterns also exhibit type-I band alignment. It should be noted that the type-I heterostructure is suitable for applications requiring efficient electron–hole recombination, such as light-emitting devices and laser applications, while the type-II heterostructure with its spatial separation of electrons and holes, is ideal for optoelectronic applications such as energy conversion and photocatalysis. All these findings indicate that controlling the stacking orders in the GeH/SiSb heterostructure allows for the fine-tuning of its electronic properties, making it versatile for various next-generation optoelectronic applications with low operational energy cost.^42^

**Fig. 6:**
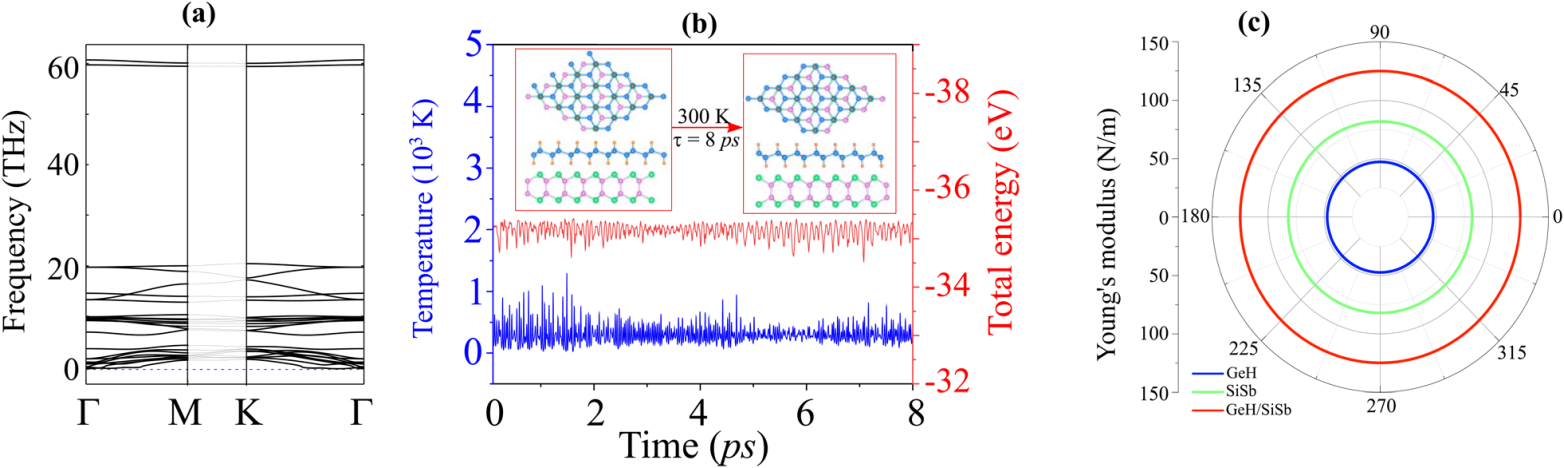
(a) The phonon spectrum, (b) fluctuations in the total energy and temperature and (c) Young's modulus of the GeH/SiSb heterostructure for the most favorable pattern.

Furthermore, we assess the stability of the GeH/SiSb heterostructure by calculating the binding energy (*E*_b_), calculating the phonon spectrum, performing AIMD simulations, and examining the mechanical behavior. The binding energy is defined as *E*_b_ = (*E*_H_ − ∑*E*_S_)/*A*, where *E*_H_ denotes the total energy of the GeH/SiSb heterostructure. *E*_S_ is the total energy of the isolated semiconductor. *A* represents the area of the heterostructure supercell. The obtained *E*_b_ for the GeH/SiSb heterostructure is listed in Table 1. The negative values of the obtained *E*_b_ confirm that all patterns of the GeH/SiSb heterostructure are stable. Moreover, these values of the *E*_b_ are comparable with those in other typical vdW systems, such as graphite^43^ and GeH-based heterostructures.^28,29^ Notably, the AB1 pattern exhibits the lowest *E*_b_, indicating that it is the most favorable configuration. Hence, all the subsequent calculations will be performed using this pattern. The phonon spectrum of the AB1 pattern in Fig. 6(a) reveals the absence of imaginary frequencies, indicating that this stacking pattern is dynamically stable. The AIMD simulations for the AB1 pattern, illustrated in Fig. 6(b), demonstrate minimal fluctuations in total energy and temperature. Moreover, the GeH/SiSb heterostructure exhibits no significant atomic distortions even after being subjected to heating up to 8 ps. These findings, combined with the minimal fluctuations in total energy and temperature, confirm the thermal stability of the GeH/SiSb heterostructure at room temperature. We further check the mechanical stability of the GeH/SiSb heterostructure by examining the elastic constants by solving the following matrix:
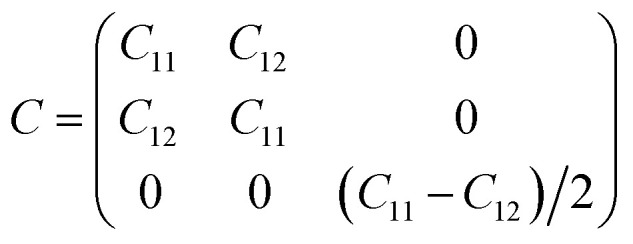
The GeH/SiSb heterostructure contains only two independent elastic constants: *C*_11_ = *C*_22_ and *C*_12_. Our calculations indicate that these elastic constants are *C*_11_ = 131.643 N m^−1^ and *C*_12_ = 30.03 N m^−1^, which satisfy the Born–Huang criterion,^44,45^*i.e. C*_11_ > *C*_12_ and *C*_66_ = (*C*_11_ − *C*_12_)/2 > 0, confirming the mechanical stability of the GeH/SiSb heterostructure. Additionally, we calculate the Young's modulus to further evaluate the mechanical properties of this heterostructure. The Young's modulus in the SiH/γ-GeSe heterostructure is measured to determine its stiffness and elasticity, providing insights into the material's ability to withstand mechanical stress without deforming:1
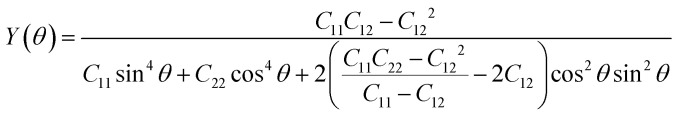


The dependence of the polar Young's modulus of the GeH/SiSb heterostructure on the angle is shown in Fig. 6(c). Notably, the combination of GeH and SiSb monolayers results in an enhanced Young's modulus for the GeH/SiSb heterostructure, indicating improved mechanical strength compared to the individual monolayers.

**Fig. 7:**
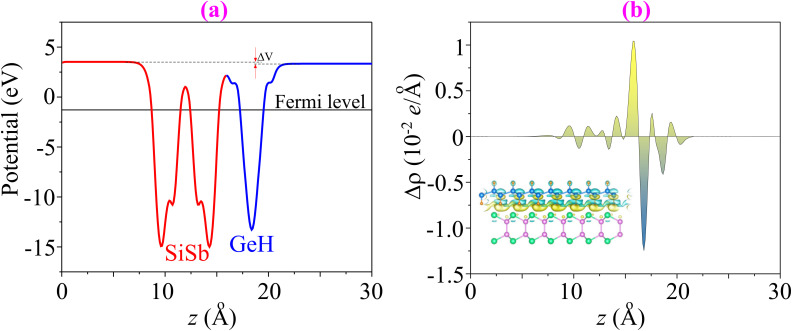
(a) Electrostatic potential and (b) charge density difference of the GeH/SiSb heterostructure. The 3D isosurface of the GeH/SiSb heterostructure is displayed in the inset, where the accumulation and depletion of charges are visualized by yellow and cyan colors.

Furthermore, to examine the charge redistribution in the GeH/SiSb heterostructure, we calculate the electrostatic potential and charge density difference (CDD). It should be noted that interlayer charge transfer at the interface of the heterostructure can still occur due to the differences in electronegativity, even though the monolayers are bonded by weak vdW interactions. This transfer impacts the electronic properties of the heterostructure, leading to unique behaviors that can be harnessed for various advanced technological applications. The SiSb layer has a lower potential than the GeH layer, leading to the formation of an interfacial dipole, as illustrated in Fig. 7. This dipole affects the charge distribution at the interface, creating a built-in electric field that influences the electronic properties of the GeH/SiSb heterostructure. The interfacial dipole drives electrons from the GeH layer to the SiSb layer while the built-in electric field is directed in the opposite direction, *i.e.*, from the SiSb to the GeH layer. This electron movement results in charge redistribution, which can be described by visualizing the CDD as follows:

2 Δ*ρ*(*z*) = ∫d*x*d*y*[*ρ*_GeH/SiSb_ − *ρ*_GeH_ − *ρ*_SiSb_]

here, the charge density of the GeH/SiSb heterostructure is denoted by *ρ*_GeH/SiSb_, while that of the constituent GeH and SiSb monolayers is marked by *ρ*_GeH_ and *ρ*_SiSb_, respectively. One can observe from Fig. 7(b) that electrons accumulate in the SiSb layer and deplete in the GeH layer. This results in negative charges appearing in the GeH layer, while positive charges concentrate in the SiSb layer. The electrons are driven from the depletion region in the GeH layer to the accumulation region in the SiSb layer upon the formation of the GeH/SiSb heterostructure. The charge redistribution at the interface of the GeH/SiSb heterostructure induces the formation of a built-in electric field, directed from the GeH layer to the SiSb layer. The potential difference is calculated to be Δ*V* = 0.05 eV, which is relatively small and exhibits an insignificant impact on the electronic properties and band alignment of the heterostructure.^46^

**Fig. 8:**
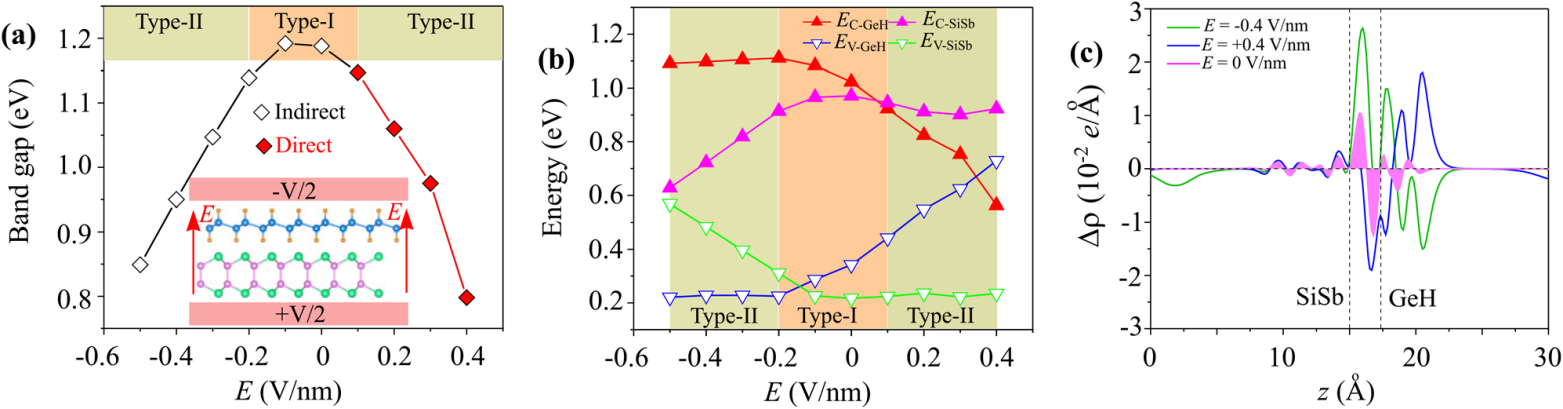
The variations in (a) the band gap of the GeH/SiSb heterostructure and (b) band edges of the GeH and SiSb layers upon the application of electric fields. (c) The planar-average CDD of the GeH/SiSb heterostructure under different strengths of applied electric fields.

Moreover, the versatility in the electronic properties of the materials is crucial for the performance of devices. It is well-established that an electric field serves as an effective strategy for tuning the electronic properties of the heterostructures. In addition to electric field tuning, mechanical strain can significantly modulate the electronic properties and contact characteristics of heterostructures.^41,47,48^ Consequently, we try to examine the effects of an applied electric field on the electronic properties of the GeH/SiSb heterostructure. The electric field directed from the SiSb layer to the GeH layer is defined as positive, as illustrated in the inset of Fig. 8(a). Firstly, it is evident that the electric field can alter the band gap of the GeH/SiSb heterostructure. The band gap of the GeH/SiSb heterostructure is generally reduced with the application of both positive and negative electric fields. This reduction makes the GeH/SiSb heterostructure highly versatile for various optoelectronic applications. The underlying mechanism of such change can be described as follows: (1) when a positive electric field is applied, it aligns with the built-in electric field, resulting in an enhancement of the total electric field, leading to a decrease in the band gap of the GeH/SiSb heterostructure. (2) With the application of the negative electric field, which opposes the built-in electric field, the total electric field is initially reduced. At a low strength of the negative electric field, below −0.1 V nm^−1^, the band gap of the GeH/SiSb heterostructure remains almost unchanged. As the strength of the negative electric field continues to increase, surpassing −0.1 V nm^−1^, the total electric field becomes stronger, leading to a reduction in the band gap. Secondly, the electric field induces a transition in the band alignment of the GeH/SiSb heterostructure between type-I and type-II. The type-I alignment, where both the band edges reside in the same layer, is ideal for devices requiring efficient electron–hole recombination, such as lasers and LEDs. Conversely, the type-II alignment, where the VBM and CBM are in different layers, is beneficial for applications that require efficient charge separation, such as solar cells and photodetectors. Thirdly, the electric field leads to a transition from an indirect to a direct band gap. The shift from an indirect to a direct band gap under the influence of an electric field further enhances the efficiency of optoelectronic devices, as direct band gaps allow for more efficient electron–hole pair recombination, improving the performance of light-emitting and absorbing devices.

**Fig. 9:**
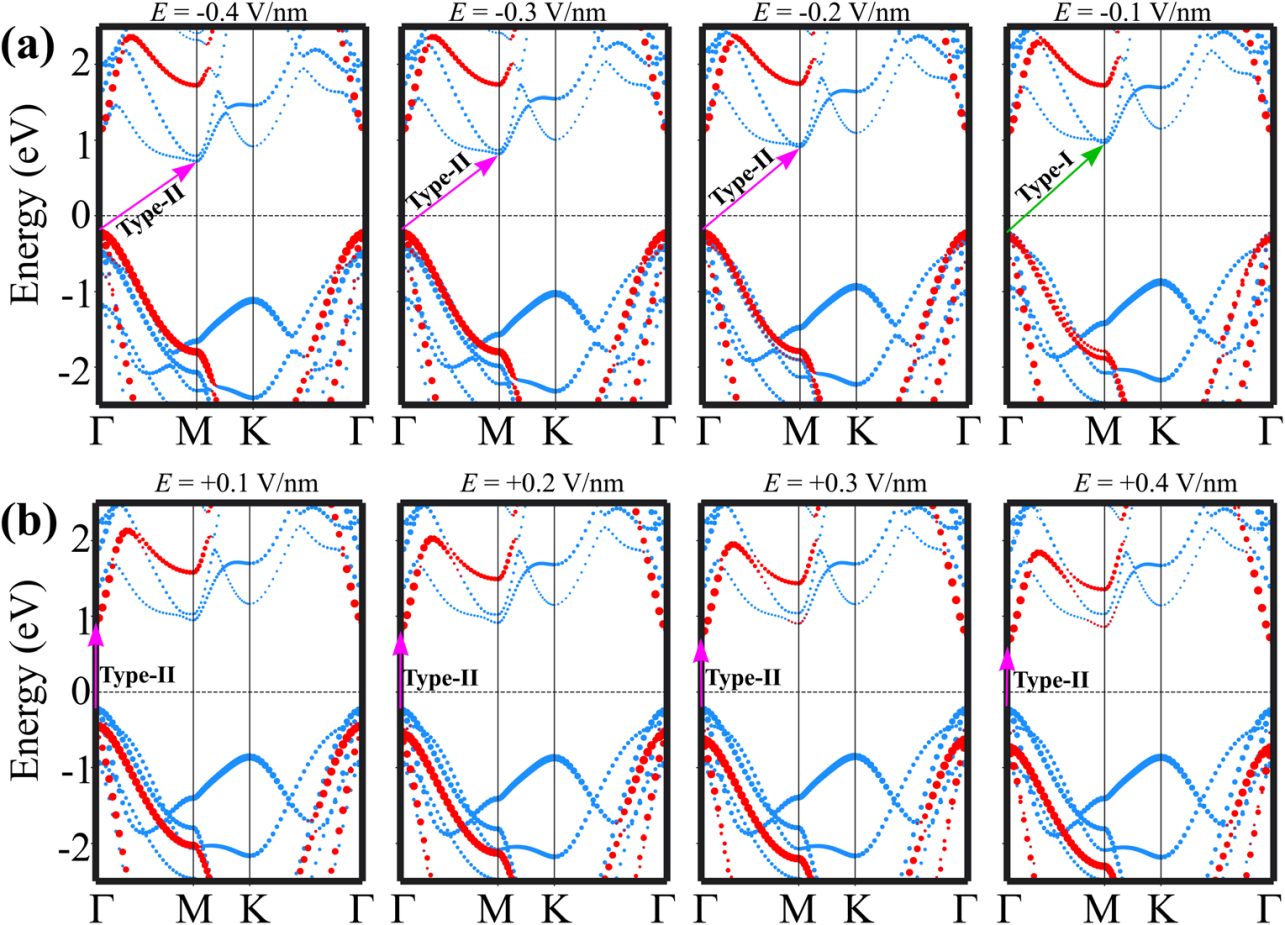
(a) Weighted projections of the band structures of the GeH/SiSb heterostructure under (a) negative and (b) positive electric fields. The weighted projections of the GeH and SiSb layers are denoted by red and blue circles, respectively. Green and purple arrows represent the type-I and type-II band alignments of the heterostructure.

The underlying mechanism of the transition between type-I and type-II band alignments, as well as the shift from an indirect to a direct band gap, can be elucidated by analyzing the positions of the band edges of the GeH and SiSb layers, the projected band structures of the GeH/SiSb heterostructure and the changes in the charge transfer difference between the two layers. The band edges relative to the Fermi level of the GeH and SiSb layers are illustrated in Fig. 8(b). With the application of a positive electric field, the energies of the VBM of the SiSb layer (*E*_V–SiSb_) and the CBM of the GeH layer (*E*_C–GeH_) remain largely unchanged. However, the energies of the VBM of the GeH layer (*E*_V–GeH_) and the CBM of the SiSb layer (*E*_C–SiSb_) shift in opposite directions. Specifically, *E*_V–GeH_ increases while *E*_C–SiSb_ decreases. Under a positive electric field of +0.1 V nm^−1^, *E*_C–GeH_ decreases to a level lower than *E*_C–SiSb_. This reduction indicates a transition from type-I to type-II band alignment. The fluctuations in the charge density differences of the GeH/SiSb heterostructure under different electric fields are depicted in Fig. 8(c). It is evident that the fluctuations in the charge density difference curves exhibit opposite trends under negative and positive electric fields. Under a negative electric field, increased electron transfer from the GeH to the SiSb layer leads to a downshift in the band edges of SiSb and an upshift in the band edges of GeH. In contrast, under a positive electric field, electron transfer is reversed, with more electrons shifting from the SiSb layer to the GeH layer. This results in a downshift of the band edges of GeH and an upshift of the band edges of the SiSb layer. Indeed, under a positive electric field of +0.1 V nm^−1^ displayed in Fig. 9(b), the CBM of the GeH layer at the *Γ* point becomes lower than that of the SiSb layer at the *M* point. This specifies a shift from an indirect to a direct band gap and confirms the transition from type-I to type-II band alignment. A transition from an indirect to a direct band gap semiconductor in the GeH/SiSb heterostructure enhances carrier recombination efficiency. Consequently, this heterostructure holds promise for optoelectronic applications, including light-emitting diodes (LEDs), lasers, and photodetectors. Conversely, when a negative electric field is applied, the band edges, including both the VBM and CBM of the GeH layer, are upshifted. In contrast, the band edges of the SiSb layer shift downward. Under a negative electric field of −0.2 V nm^−1^, as illustrated in Fig. 9(a), the VBM of the GeH layer becomes higher than that of the SiSb layer, indicating a transition from type-I to type-II band alignment. Therefore, applying an electric field induces not only a change in the band gap of the GeH/SiSb heterostructure but also transitions between type-I and type-II band alignments and shifts from an indirect to a direct band gap. These findings could provide valuable insights for designing next-generation optoelectronic devices based on the GeH/SiSb heterostructure, enabling fine-tuning of their electronic properties for optimized performance across various applications.

## 4 Conclusions

In this study, we comprehensively investigated the GeH/SiSb heterostructure, focusing on its intrinsic properties, stability, and tunability under different stacking patterns and electric fields. We found that the combination of these monolayers leads to a stable heterostructure, as confirmed by phononic spectrum analysis, AIMD simulations, and mechanical property evaluations. Our results demonstrated that the GeH and SiSb monolayers can form either type-I or type-II band alignments depending on the stacking patterns. Furthermore, we observed that the electronic properties of the GeH/SiSb heterostructure are highly tunable. The application of an electric field can modulate the band gap, induce transitions between type-I and type-II band alignments, and shift the band gap from indirect to direct. Our insights highlight the significant potential of the GeH/SiSb heterostructure in next-generation optoelectronic applications, such as photodetectors and solar cell applications.

## Data availability

The data that support the findings of this study are available from the corresponding author upon reasonable request.

## Conflicts of interest

There are no conflicts to declare.

## Supplementary Material

NA-OLF-D5NA00251F-s001
