# Supplementary material for: First-principles exploration of the electronic versatility of the GeH/SiSb heterostructure through stacking and electric fields
Source: Nanoscale Adv. 2025 May 21;7(13):4205–13. doi: 10.1039/d5na00251f (PMC12138814; doi:10.1039/d5na00251f)
Supplement: NA-007-D5NA00251F-s001 [file NA-007-D5NA00251F-s001.pdf]

Supplementary Information (SI) for Nanoscale Advances.
This journal is © The Royal Society of Chemistry 2025

Supporting Information (SI)

Nguyen Xuan Sang[1,2], Khang D. Pham[3,4,†]

[1]*Atomic Molecular and Optical Physics Research Group, Institute for Advanced Study in Technology, Ton Duc Thang University, Ho Chi Minh City, Vietnam. Email: nguyenxuansang@tdtu.edu.vn*
[2]*Faculty of Electrical and Electronics Engineering, Ton Duc Thang University, Ho Chi Minh City, Vietnam*
[3]*Institute of Research and Development, Duy Tan University, Da Nang 550000, Vietnam. E-mail: phamdinhkhang@duytan.edu.vn*
[4]*School of Engineering & Technology, Duy Tan University, Da Nang 550000, Vietnam*

† *To whom correspondence should be addressed*

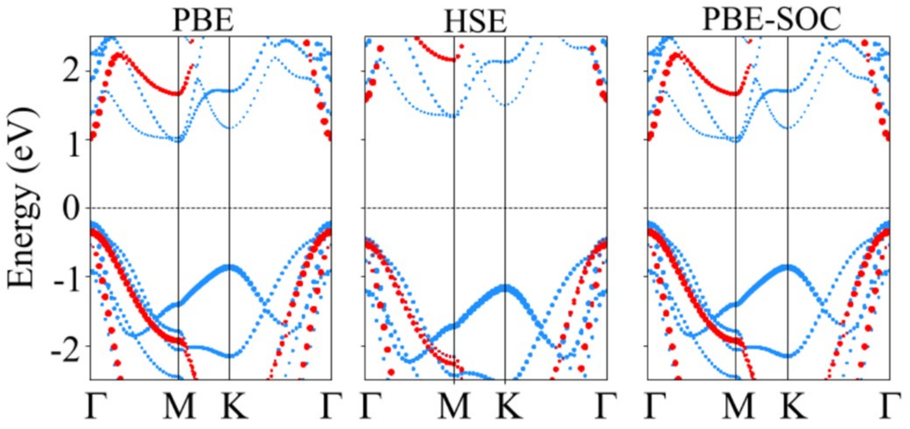

Fig. S1. Projected band structures of the GeH/SiSb heterostructure for the most favorable stacking pattern AB1 obtained by PBE, HSE and PBE-SOC methods.
